# In Situ Synthesis of Hierarchical Flower-like Sn/SnO_2_ Heterogeneous Structure for Ethanol GAS Detection

**DOI:** 10.3390/ma16020792

**Published:** 2023-01-13

**Authors:** Ye Zhu, Li Yang, Shenghui Guo, Ming Hou, Yanjia Ma

**Affiliations:** 1 State Key Laboratory of Complex Nonferrous Metal Resources Clean Utilization, Kunming University of Science and Technology, Kunming 650093, China; 2 Faculty of Metallurgical and Energy Engineering, Kunming University of Science and Technology, Kunming 650093, China

**Keywords:** in situ synthesis, flower-like, Sn/SnO_2_, gas sensor, ethanol

## Abstract

In this study, morphogenetic-based Sn/SnO_2_ graded-structure composites were created by synthesizing two-dimensional SnO sheets using a hydrothermal technique, self-assembling into flower-like structures with an average petal width of roughly 3 um. The morphology and structure of the as-synthesized samples were characterized by utilizing SEM, XRD, XPS, etc. The gas-sensing characteristics of gas sensors based on the flower-like Sn/SnO_2_ were thoroughly researched. The sensor displayed exceptional selectivity, a rapid response time of 4 s, and an ultrahigh response at 250 °C (Ra/Rg = 17.46). The excellent and enhanced ethanol-gas-sensing properties were mainly owing to the three-dimensional structure and the rise in the Schottky barrier caused by the in situ production of tin particles.

## 1. Introduction

In addition to its conventional use in the wine and pharmaceutical industries, ethanol has recently received a great deal of attention in the energy sector. For instance, using ethanol gasoline as a fuel blend to enhance engine efficiency and reduce traditional emissions in cars has produced significant benefits [1,2,3]. As a result, the question of how to create an optimal gas sensor with a remarkable response time has gained importance.

Controlling the shape and size of metal oxide semiconductor gas-sensitive materials has received a lot of interest lately [4,5,6]. SnO_2_ is one of the semiconductor gas sensors with the most research and the widest variety of applications [7,8,9,10]. It demonstrates excellent gas-sensitive properties for various poisonous and hazardous gases. However, there is still much room for improvement in the existing sensitivity and selectivity of SnO_2_ gas sensors, which cannot yet match the needs of field environmental detection [11].

The demands of gas sensor applications can no longer be satisfied by a single SnO_2_ material. The right noble and transition metals can be added to the SnO_2_ sensor to increase its selectivity and sensing responsiveness [12,13]. These actions of metals cooperate with the semiconductor properties of the SnO_2_ sensor [14]. Xu et al. have synthesized Pr-doped SnO_2_ hollow beaded tubular nanostructure with the electrospinning technique; the material showed excellent sensitivity and selectivity toward ethanol. The material recovered quickly (2 s) to 100 ppm ethanol at 200 °C but took awhile to respond (8 s) [15]. Meng et al. fabricated a Pd@Pt/SnO_2_ hybrid-based gas sensor for the detection of hydrogen at near-ambient temperatures. Although the material may function at near-ambient temperatures, its manufacture requires a wet chemical method that is time-consuming (more than 47 h), difficult, and expensive because Pd and Pt are both precious metals [16]. As far as we know, all these previous studies showed different results on sensing but no one reported the hierarchical flower-like Sn/SnO_2_ materials as a gas sensor.

Tin oxide has so far been devised and synthesized using a variety of three-dimensional (3D) structures for the detection of different gases and has demonstrated good sensing capabilities. Because the unique 3D hierarchical flower-shaped structures have more surface-active areas, higher surface permeability, and less agglomeration, they help improve the gas sensing performance. Ma et al. created SnO_2_/ZnO heterostructures with pores resembling flowers from SnS_2_/ZIF-8 using just a basic template approach and subsequent heat treatment process. In comparison to pure tin oxide sensors, the SnO_2_/ZnO heterostructures demonstrated noticeably better sensing capability for triethylamine (TEA) gas at 50 ppm with a response of around 17.7 at an operating temperature of 200 °C [17]. The material displayed a gas-sensitive response value of 13.5 to ethanol at 500 °C to 100 ppm when Wei et al. prepared Ag/SnO_2_ nanoflower-like structures using a conventional impregnation method in which Ag nanoparticles of 20–30 nm size were uniformly grown onto the surface of tin oxide products [18]. The three-dimensional layered WO_3_-SnO_2_ nanoflower-like (NFs) composite was effectively created by XUE et al. using a hydrothermal synthesis of tungsten trioxide and tin oxide as precursors. According to the findings of the gas sensitivity tests, the WO_3_-SnO_2_ nanocomposite outperforms pure tin oxide at methane detection. The WO_3_-SnO_2_ based sensor was 2.3 times more sensitive to 500 ppm methane (S = 2.85) than the pure SnO_2_-based sensor at this temperature [19], which decreased the optimal working temperature of the SnO_2_-based sensor from 120 °C to 110 °C. To our knowledge, however, there have not been many attempts to investigate novel ethanol-gas-detecting nanomaterials by combining layered flower-shaped nanostructures with the doping of Sn ions, despite the fact that doing so would be extremely beneficial.

Inspired by the above studies, we have successfully synthesized Sn/SnO_2_ flower-like structure composites through hydrothermal synthesis, followed by heat treatment, and their microstructures were characterized. Their morphology, nanostructures, chemical components, and gas-sensing properties, mainly ethanol, were studied. The results show that the sensitivity of the ethanol sensor was significantly improved by synthesizing the 3D hierarchical structures to increase the specific surface area and by modifying the surface of the structures with metals to enhance the interaction of the adsorbed gas. This work has been providing a new exploration direction for semiconductor materials for detecting ethanol. This work illustrates the relationship between the internal composition, structure, and function of flower-like Sn/SnO_2_ multistage composite gas-sensitive materials, providing a simple and effective strategy for the development and design of high-performance ethanol-gas-sensitive materials.

## 2. Materials and Methods

**Figure 1:**
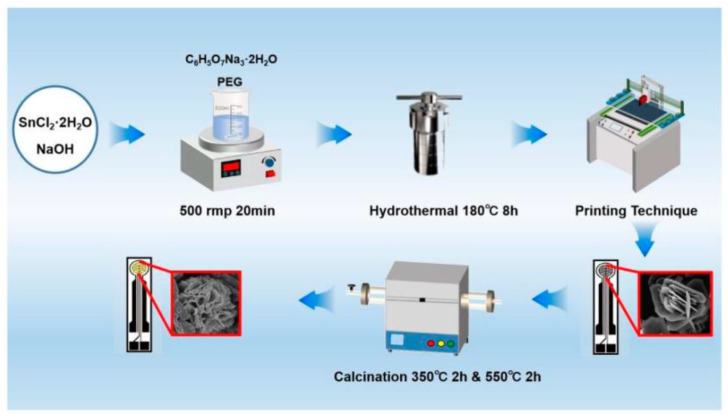
Schematic illustrating for the formation process of Sn/SnO_2_ sample.

Synthesis of flower-like Sn/SnO_2_ materials: 2 mmol of stannous chloride (SnCl_2_-2H_2_O) was dissolved in 40 mL of deionized water and magnetically stirred until completely dissolved. Weigh 4 mmol sodium hydroxide (NaOH), 0.34 mmol sodium citrate (C_6_H_5_O_7_Na_3_-2H_2_O), and 0.1 mmol PEG (MW = 600); dissolve them in the above SnCl_2_ solution, and stir magnetically for 10 min to obtain a clear solution. The produced solution was put into a 50 mL Teflon reactor, where it reacted for 8 h at 180 °C before cooling naturally. The gray–black SnO precipitate underwent numerous washes with anhydrous ethanol before being dried at 60 °C in a vacuum drying oven after being neutralized with deionized water. The obtained black powder was placed in a tube furnace and sintered at 350 °C for two hours under argon gas protection and then continued to sinter at 550 °C for two hours to obtain Sn/SnO_2_ samples. The schematic illustration of sample preparation is shown in Figure 1.

X-ray diffraction (Rigaku D/max-2500, Tokyo, Japan) was used to examine the crystal structures of the materials in their as-prepared state using Cu Kα radiation (λ = 1.5418 Å) in the 2θ range from 5° to 90°. The samples’ microstructures were examined using a field-emission scanning electron microscope (Hitachi S-4500, Tokyo, Japan). X-ray photoelectron spectroscopy was used to analyze the particles’ chemical makeup and elemental valence state (PHI5000 VersaProbe II, Tokyo, Japan).

The platinum electrode used in the gas-sensing device comprised a heating electrode and a measuring electrode. The samples were prepared for the gas-sensing studies using the following procedures: First, a platinum sizing agent was screen-printed onto the aluminum oxide (Al_2_O_3_) substrate (6 × 30 mm) to prepare it for the measuring electrode transducer. The Al_2_O_3_ matrixes were subsequently dried in an oven at 70 °C for 30 min before being sintered for 20 min at 350 °C and then at 850 °C. An electrode chip was used to screen-print the sample pastes onto the Al_2_O_3_ matrix, creating a sensing film with a thickness of 5–10 μm. The sensing film was subsequently dried for 40 min at 70 °C [20].

To increase the mechanical bond strength of the particles in the films, the as-prepared samples were annealed at 400 °C for 2 h and then continued to sinter at 550 °C for 2 h under an argon environment. After calcination, the samples were collected and preserved for later use. In the dry air, they were heated to 300 °C using a heating electrode for 24 h aging.

An industrial device for assessing the performance of gas sensors called the SD-101 (Huachuang Ruike Tech. Co., Ltd., Wuhan, China), which can test four gas sensors simultaneously, was used to evaluate the effectiveness of the gas sensors. During the testing of the gas sensors, the power supply of the heater coil was automatically changed by a microcontroller to reach temperatures ranging from 150 °C to 450 °C at 10 V of voltage. At a concentration of 1000 ppm (*v*/*v*), volatile gases such as acetone, methanol, ethanol, and formaldehyde were tested using a static method.

At temperatures of 150 °C, 200 °C, 250 °C, and 300 °C, respectively, the sensors were functional. The ambient temperature and relative humidity for the duration of the testing were 18–20 °C and 75–80%, respectively. Using ethanol as an example, the following was a common test procedure: (1) until the resistances of the gas sensors stabilized in air, the sensors were mounted on the SD-101 device and exposed to air in a 50 L chamber for 5 min at a constant operating temperature; (2) a microinjector was used to inject the appropriate amount of ethanol into the chamber’s heating panel, and two fans circulated the vaporized ethanol throughout the chamber. (3) After the fans were turned off to maintain static air in the chamber, the resistances of the sensors stabilized once more; (4) the sensors recovered before the following measurement when the chamber was opened and fresh air was added. At least three devices were evaluated for each type of sensor, and the average results are shown. The gas sensor’s response was computed as [21]:

(1) $$S\;=\frac{R_{a}}{R_{g}}$$

where R_a_ denotes the resistance in dry air, and R_g_ denotes the resistance in test gas.

**Figure 2:**
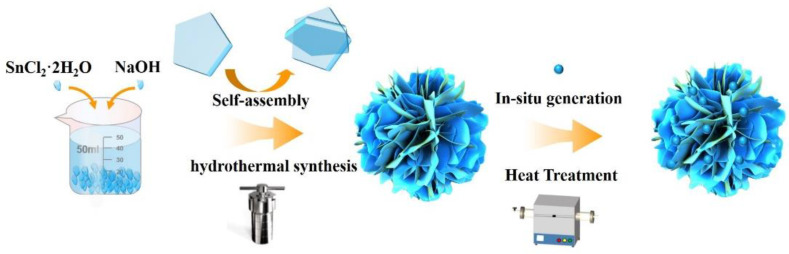
Schematic diagram of the preparation of flower-like Sn/SnO_2_ composites.

Figure 2 shows the preparation of Sn/SnO_2_ composites. As shown in Figure 2, with the hydrothermal reaction, the flake SnO is formed first in the solution, and the flake structure gradually self-assembles into a three-dimensional flower-like material with time delay. In addition, the Sn/SnO_2_ flower-like heterogeneous structure was formed by annealing.

## 3. Results

Material Characterization

**Figure 3:**
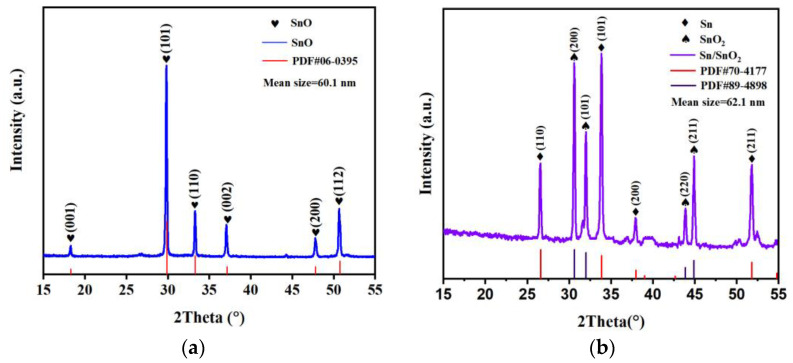
XRD patterns of (**a**) SnO precursor; (**b**) Sn/SnO_2_.

The composition of the physical phases of the composite precursors and final samples was analyzed by XRD. Figure 3a shows the XRD pattern of the composite precursors, and in Figure 3a, we observe the diffraction peaks of SnO (PDF06-0395), wherein the strongest diffraction peak appears at 29.8°, corresponding to the (101) crystal plane. Figure 3b shows the XRD patterns of the prepared composites, in which we observe distinct diffraction peaks of SnO_2_ (PDF70-4177) and Sn (PDF89-4898) at 33.9°, corresponding to the (101) crystal plane of SnO_2_, and 30.6°, corresponding to the (200) crystal plane of Sn. The results indicate that the main phase of the composite precursor prepared by the hydrothermal method was SnO, and after roasting treatment, the final gas-sensitive material consisting of Sn and SnO_2_ phases was obtained. Among them, the reaction of SnO roasting at 350–550 °C can be represented by the following chemical equation.

(2) $$4{\mathrm{SnO}}_{\left(s\right)}\to {\mathrm{Sn}}_{3}O_{4}{}_{\left(s\right)}+{\mathrm{Sn}}_{\left(l\right)}$$

(3) $${\mathrm{Sn}}_{3}O_{4}{}_{\left(s\right)}\to 2{\mathrm{SnO}}_{2}{}_{\left(s\right)}+{\mathrm{Sn}}_{\left(l\right)}$$

We calculated the crystallite size of the material with the Debye–Scherrer Equation (4), and the results showed that the crystallite size of the composite increased after annealing, but we found no significant change in the morphology of the composite before and after annealing by SEM mapping. This indicates that the size of the grain has no significant effect on the microstructure of the composite.

(4) D =0.943λ/βcosθ

where λ is wavelength of the X-rays; β is the full width at half maximum (FWHM) of the diffraction peak; θ is the angle of the diffraction, and D is the crystallite size.

**Figure 4:**
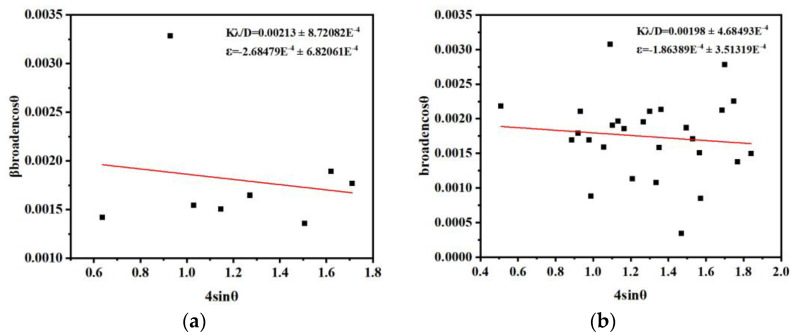
Microscopic strain diagram of (**a**) SnO precursor; (**b**) Sn/SnO_2._

We also calculated the microscopic strain of the composite according to the Williamson–Hall Equation (5). From Figure 4, we can see that the microscopic stress after annealing did not cause significant microscopic strain, which resulted in no significant change in the microstructure of the annealed Sn/SnO_2_ flowers.

(5) $$\beta_{\mathrm{broaden}}\cos\theta \;=\frac{k\lambda }{D}+4\varepsilon \sin\theta$$

where D is the grain size; θ is the radian value of the peak position; λ is the wavelength; k is a constant; β broaden is the total spread, and ε is the microscopic stress.

**Figure 5:**
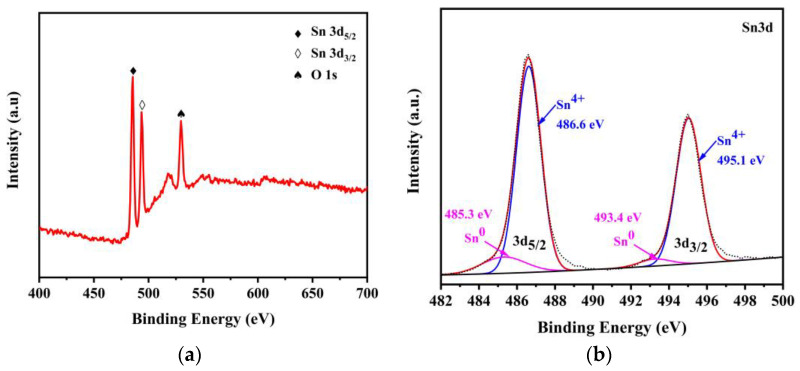
XPS spectrum of Sn/SnO_2_ sample.

In order to investigate the elemental composition of the Sn/SnO_2_ sample and the chemical valence of each element, it was characterized by XPS, and the results are shown in Figure 5. The XPS spectra demonstrate that the sample consists of Sn and O elements, and Figure 5a shows a high-resolution spectrum at O 1s, with a peak occurring around 531 eV. The Sn 3d5/2 and Sn 3d3/2 spectra, shown in Figure 5b, have a dominant peak at 486.6 eV and 495.0 eV, both corresponding to tin oxide, while the half-peak width of the double peak indicates the presence of more than one chemical state, with shoulder peaks at 485.3 eV and 493.4 eV corresponding to the metal Sn. Combined with the XRD results, it can be further inferred that SnO undergoes disproportionation reactions to form Sn and SnO_2_ under high-temperature roasting.

**Figure 6:**
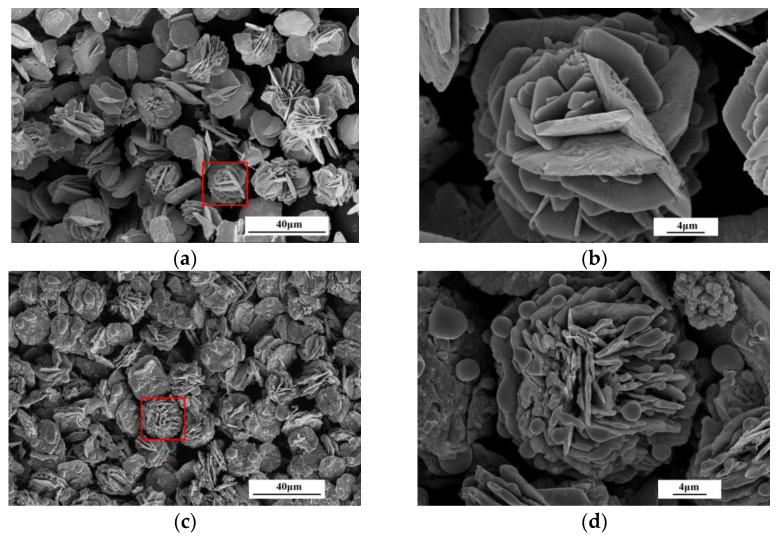
FESEM images of microstructure of (**a**,**b**) SnO precursor, (**c**,**d**) Sn/SnO_2_ sample.

The morphological analysis of the obtained samples is shown in Figure 6a,c. It shows that the prepared SnO precursors are composed of two-dimensional sheet materials mosaicked with each other to form a flower-like structure, wherein the size of the two-dimensional sheet material is 3 μm, and is more uniformly dispersed. The advantage of this flower-like structure over the common sheet structure is that the larger specific surface area is beneficial in providing more active sites and improving the gas-sensitive response characteristics. Figure 6b,d shows the Sn/SnO_2_ composites produced after roasting the flower-like SnO precursors. From the figures, it can be seen that the morphology of the Sn/SnO_2_ composites obtained after roasting maintains the original morphology of the SnO precursors, showing peony-flower-like and sheet-like structures, but the morphology reduces the thickness of petals and increases the number of petals. There may be two reasons for this. Firstly, it is known from Equations (2) and (3) that the reaction to obtain the final product from the precursor is a thermal decomposition reaction. Then, the volatilization of the organic slurry added during the device preparation process during the heat treatment may cause the fine gaps as a precursor to expanding open. Furthermore, the petals are distributed with particles of different sizes.

**Figure 7:**
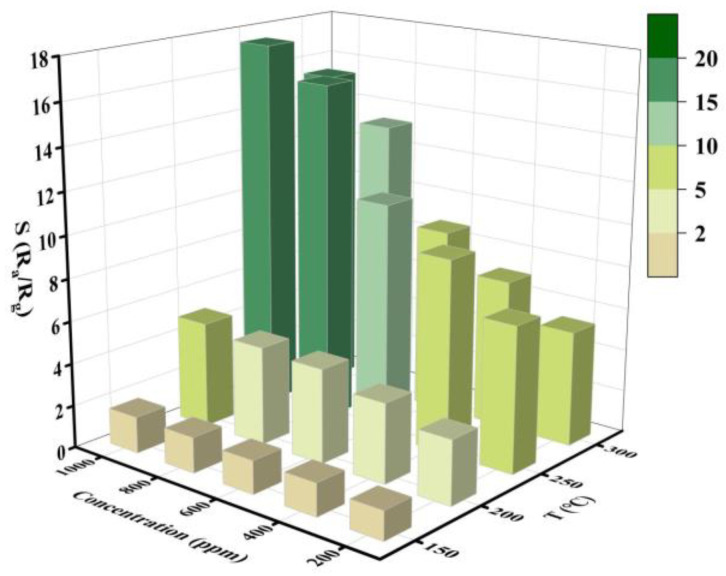
Gas-sensing response of Sn/SnO_2_ sample to different ethanol concentrations at different operating temperatures.

The metal oxide semiconductor gas sensor is significantly influenced by the operating temperature of the gas sensor [22,23,24,25]. As shown in Figure 7, the response to various concentrations of ethanol was evaluated at various operating temperatures after it was manufactured as a gas-sensitive element in order to establish the sample’s ideal working temperature. It may be determined that 250 °C is the ideal operating temperature for the gas-sensitive element based on Figure 7’s depiction of the response value of the element when the operating temperature increases from 200 to 400 °C.

Meanwhile, Figure 7 depicts the response of the sample’s gas-sensitive element to various ethanol concentrations. Figure 7 shows that the response value of gas-sensitive elements gradually increased with increasing ethanol concentration, reaching 17.46 when the methanol concentration reached 1000 ppm.

**Figure 8:**
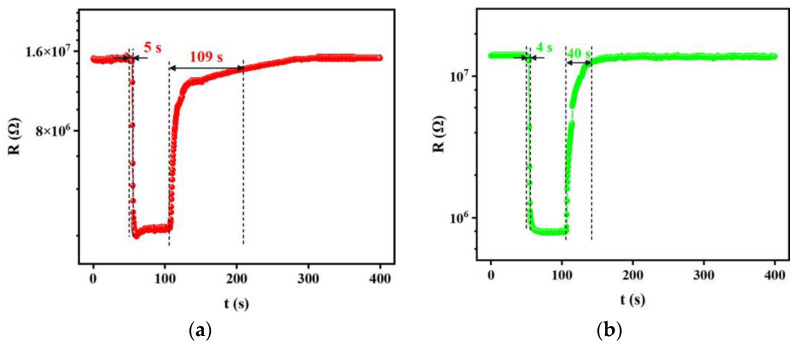

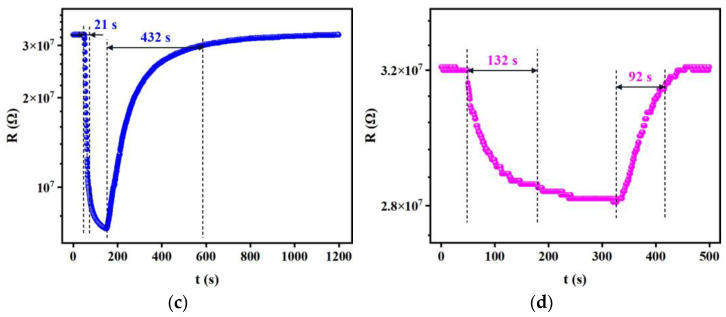
Recovery curves of the response of Sn/SnO_2_ samples to different VOCs at 1000 ppm at 250 °C. (**a**) Methanol, (**b**) ethanol, (**c**) formaldehyde, and (**d**) acetone.

The selectivity of the gas sensor is critical in practical applications [26,27,28,29,30]. Figure 8 shows that, among all gases, the response time of the sample gas-sensing element for ethanol gas is 4 s, which is 4/5, 4/21, and 1/33 of the response time for methanol, formaldehyde, and acetone, respectively. At the same time, the recovery time of the sample gas-sensing element for ethanol gas is 40 s, which is 40/109, 5/54, and 10/23 of the response recovery time for methanol, formaldehyde, and acetone, respectively.

**Figure 9:**
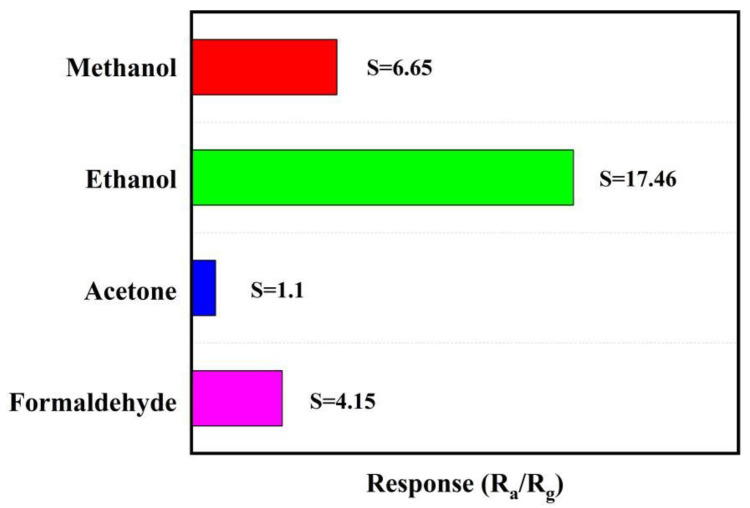
Gas response of Sn/SnO_2_ sample to 1000 ppm of different VOCs at 250 °C.

Meanwhile, Figure 9 depicts the gas-sensitive response values of the sample to 1000 ppm of methanol, ethanol, formaldehyde, and acetone at 250 °C are 6.65, 17.46, 1.1, and 4.15, respectively. it is obvious that the sample has the best selectivity for ethanol.

**Figure 10:**
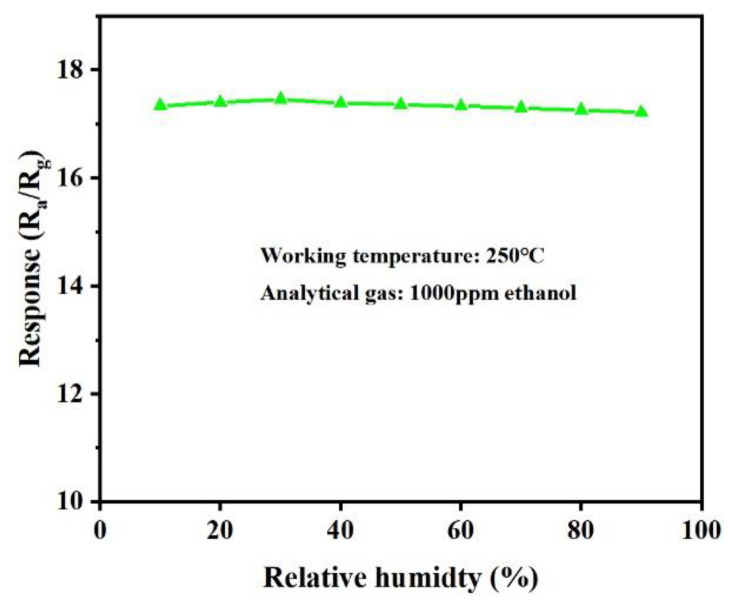
Sensing response at different RH levels for ethanol concentrations of 1000 ppm at 250 °C.

**Figure 11:**
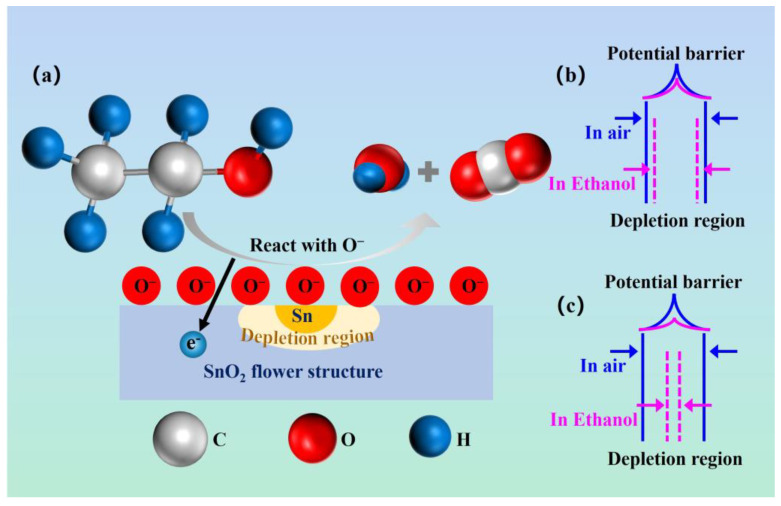
Schematic diagram of the possible gas-sensing mechanism of Sn/SnO_2_ samples. (**a**) Schematic diagram of gas sensing mechanism of gas sensor, (**b**,**c**) The energy band structure of pure and Sn-doped SnO_2_.

As shown in the Figure 10 and Figure 11, the sensing response of the sensor of the Sn/SnO_2_ flower-like heterogeneous structure to 1000 ppm ethanol was also measured at different relative humidity (RH) environments. It was found that the gas response increased slightly at the RH level of 10–30% and then decreased with a further increase in RH. This may be due to competition between chemisorbed oxygen species and water molecules adsorbed on the sensor surface. Although there was some decrease in response at high humidity, it was not significant, indicating that it can be used in high-humidity environments.

To highlight the advantages of this work, we investigate the relevant gas-sensitive materials as shown in Table 1. In this work, we obtain an ethanol gas-sensitive sensor with excellent response speed by constructing a flower-like Sn/SnO_2_ multistage heterostructure.

The gas-sensitive properties of the morphogenetic-based Sn/SnO_2_ multistage semiconductor interface are demonstrated by the performance test results. The gas-sensing mechanism of metal oxide gas sensors is related to the number of adsorption sites and oxygen atoms on the surface. Due to the hierarchical structure of Sn/SnO_2_, a very large volume and specific surface area exist. The presence of a large number of surface-adsorbed oxygen with dangling bonds on the material surface can be absorbed by ethanol molecules and extracted from the Sn/SnO_2_. This process can lead to the release of electrons from Sn/SnO_2_, which results in a change in the Fermi energy level. When Sn/SnO_2_ is exposed to ethanol vapor, oxygen ions react with ethanol molecules and transfer a large number of electrons to the grains. These additional carriers reduce the distance between the Fermi energy level and the conduction band, thus increasing the electrical conductivity of the sample [36].

The gas-sensitive principle-diagram of the morphogenetic Sn/SnO_2_ multistage composite is shown in Figure 8. The redox reaction mechanism is commonly used to explain the sensitivity mechanism of metal oxide SnO_2_. SnO_2_ materials are N-type semiconductor materials with a broad bandgap (3.6 eV), and, under normal conditions, the internal O 1s are usually composed of two types of oxygen; one is lattice oxygen (O_lat_), and the other is adsorbed oxygen (O_ads_) on the surface [37,38]. In the air, ion-adsorbed oxygen generates spatially induced charges on the semiconductor surface and creates surface potential barriers that bend the energy band upward, resulting in a decrease in the surface mobility of the SnO_2_ material due to the decrease in the electron concentration after the adsorption of oxygen. Below 100 °C, the type of O_ads_ on the SnO_2_ surface is mainly O_2_^−^. At an operating temperature of 250 °C, the O_ads_ of the SnO_2_ gas-sensitive element is mainly in the form of O^−^_ads_. The adsorption process of composites is closely related to the loading of the in situ-generated Sn particles. The influence has the role of Schottky junctions formed at the oxygen on the surface of SnO_2_ that can be expressed as [39,40,41,42]:

O_2(gas)_ → O_2(ads)_ (6)

O_2(ads)_ + e^−^ → O_2_^−^_(ads)_ (7)

O_2_^−^_(ads)_ + e^−^ → 2O^−^_(ads)_ (8)

At 250 °C, oxygen molecules adsorb electrons based on morphogenetic Sn/SnO_2_ hierarchical composites to form oxygen anions (O^−^). A region of electron depletion is formed on the surface of the semiconductor material, and the surface energy band is bent, increasing the resistance of the material. After the SnO_2_ sensor is exposed to ethanol, the reaction between the oxygen anion and the ethanol gas releases electrons. These electrons then return to the surface of the semiconductor material, reducing the thickness of the surface depletion region, reducing the energy band bending, and decreasing the resistance.

The Sn particles will facilitate the reactivity of the oxygen anion deposited on the surface of tin oxide with the test gas when it is added. A thinner depletion layer will arise from the oxygen anion’s trapped electrons being discharged back onto the tin oxide’s surface. The ethanol molecule reacts with the oxygen anion initially adsorbed onto the surface of the matter as follows [43,44]:

CH_3_CH_2_OH _(gas)_ → CH_3_CH_2_OH _(ads)_ (9)

CH_3_CH_2_OH _(ads)_ + 6O^−^_(ads)_ → 2CO_2_ + 3H_2_O + 6e^−^_(ads)_ (10)

The reaction between air and ethanol gas involves more oxygen molecules when Sn particles are present, increasing the sensitivity of the gas. Due to more oxygen being adsorbed on the surface of Sn in air, the Schottky barrier will rise, and the material’s resistance will rise along with the width of the crystal depletion layer. The Schottky barrier and resistance are decreased when ethanol gas is injected because of the interaction between the SnO_2_ surface and the ethanol. The Schottky barrier mechanism is the cause of the increased gas sensitivity.

## 4. Summary and Conclusions

In conclusion, using a straightforward hydrothermal process and heat treatment, we developed 3D flower-like Sn/SnO_2_ composites to obtain outstanding ethanol-sensitive materials. According to studies on sensing mechanisms, the combined impacts of 3D structure and dopant effects on the flower-shaped Sn/SnO_2_ composite sensor are key for the much-improved ethanol response. The 3D flower-like Sn/SnO_2_ composites also have good selectivity, a quick response time (4 s), and a low operating temperature (250 °C). Thus, the 3D flower-like Sn/SnO_2_ architecture is a potential sensing material for the practical detection of low ethanol concentrations.

## Figures and Tables

**Table 1:** Gas-sensing performance toward different gases reported in this work and in the literature.

Sensing Material	Detection Gas	Con. (ppm)	Tem. (°C)	Res/Rec (s)	Response	Ref.
Ce-SnO_2_ spheres	acetone	100	250	17/38	11.9	[31]
flower-like CuO	ethanol	1000	260	5/15	4	[32]
Sn/SnO_2_@NGC	NH_3_	300	R.T	148/136	142.2	[33]
SnO nanofiber-Pt	methane	1000	350	24/141	4.5	[34]
SnO_2_ hollow microspheres	HCHO	100	300	30/30	7	[35]
flower-like Sn/SnO_2_	ethanol	1000	250	4/40	17.46	this work

## Data Availability

Not applicable.
